# Patient with clinical celiac disease mimicking triple‐negative essential thrombocythemia

**DOI:** 10.1002/ccr3.5197

**Published:** 2022-04-07

**Authors:** Elrazi A. Ali, Kamran Mushtaq, Elabbass Abdelmahmuod, Mohamed A. Yassin

**Affiliations:** ^1^ Internal Medicine Department Hamad Medical Corporation Doha Qatar; ^2^ Department of Gastroenterology Hamad Medical Corporation Doha Qatar; ^3^ Department of Hematology and Oncology Hamad Medical Corporation Doha Qatar

**Keywords:** celiac disease, essential thrombocythemia, thrombocytosis, triple‐negative ET

## Abstract

Platelets are acute‐phase reactants, which can be elevated due to a secondary cause or less commonly because of a primary mechanism. Primary disorders include hematological conditions such as myelodysplastic syndrome, acute myeloid leukemia, chronic myeloid leukemia, polycythemia vera, and essential thrombocythemia (ET). Most ET patients have a mutation in the genes regulating thrombopoiesis, *JAK2*, *CALR*, or *MPL* genes. But 10%–15% of ET patients are triple‐negative, where patients have no detectable mutation. We report a young patient with no significant past medical history evaluated for persistent thrombocytosis. She was initially diagnosed as triple‐negative ET based on a bone marrow biopsy. She had positive antibodies for celiac disease, and the diagnosis was confirmed by a small bowel biopsy, which is confirmatory for diagnosing celiac disease in adults. We recommend screening triple‐negative ET patients for celiac disease before going to more expensive tests.

## Graphical abstract

Triple‐negative ET patients should be screened for celiac disease before doing more sophisticated studies such as gene sequencing or more invasive tests such as bone biopsy. This may reveal underdiagnosed celiac patients and avoid related complications.

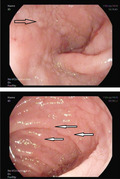

## 1 BACKGROUND

Thrombocytosis is an increased platelet count above the normal range (more than 450 10^3^/μl).^1^ Causes can be reactive (secondary) or primary. Primary causes are due to mutations in the genes regulating thrombopoiesis or due to malignant clones, as in myeloproliferative neoplasms (MPN). Secondary causes include iron deficiency anemia, chronic inflammatory conditions, infections, malignancies, rheumatological conditions, and post‐splenectomy or hyposplenism. One of the causes of reactive thrombocytosis is celiac disease, a small bowel disorder characterized by mucosal inflammation, villous atrophy, which occurs upon exposure to dietary gluten and demonstrates improvement after withdrawing gluten from the diet. The disease pattern varies from severe to subclinical or asymptomatic.^2^ The diagnosis was confirmed by the typical finding on the upper small bowel biopsy. Usually, it presents with gastrointestinal symptoms such as diarrhea, abdominal pain, and bloating. However, it can present with non‐gastrointestinal symptoms.^3^ Extra‐intestinal manifestations and complications are not related to the severity of the enteropathy.^4^, ^5^, ^6^ They include hematological manifestations such as thrombocytosis. The elevation in platelet count in celiac disease can be secondary to iron deficiency, hyposplenism, or occasionally no cause is identified.^7^ Essential thrombocythemia (ET) is a myeloproliferative neoplasm (MPN) characterized by excessive, clonal platelet production.^4^ To diagnose ET, patients must meet all four major criteria or the first three major criteria and the minor criterion of the 2016 WHO criteria for MPN. The major criteria include the demonstration of Janus Kinase 2 (*JAK2)*, *CALR*, or *MPL* mutation,^4^ which is present in approximately 90 percent of cases. However, the remaining percent who are negative for these mutations (triple‐negative disease) might have mutations in other genes^8^ or have no identified mutation.^9^ We reported this case as celiac disease presenting as a mimicker of triple‐negative ET.

## 2 CASE REPORT

We report a 32‐year‐old woman with no significant past medical history, married, and has no children, two abortions. She was evaluated for persistent thrombocytosis with platelet count in the range 460–560 10^3^/μl (150–400 10^3^/μl). Physical examination was unremarkable. Investigations revealed hemoglobin of 11.1 gm/dl (12–15 gm/dl) and ferritin level 8 mcg/L (11–304 mcg/L), hemoglobin electrophoresis showed normal Hb pattern. Peripheral blood smear showed normocytic normochromic red cells with increased rouleaux formation and within normal hemoglobin level, with increased platelets count.

She was started on intravenous iron therapy; however, thrombocytosis persisted after anemia correction. Hemoglobin improved to 12.8 gm/dl and ferritin levels rose to 252 mcg/L. Further evaluation for persistent thrombocytosis, *JAK*‐2, *CALR*, *MPL*, and *BCR ABL* negative. Chromosomal analysis revealed a normal karyotype: (46, XX). A bone marrow biopsy done showed cellular bone marrow with increased megakaryopoiesis and no increase in blasts.

**FIGURE 1:**
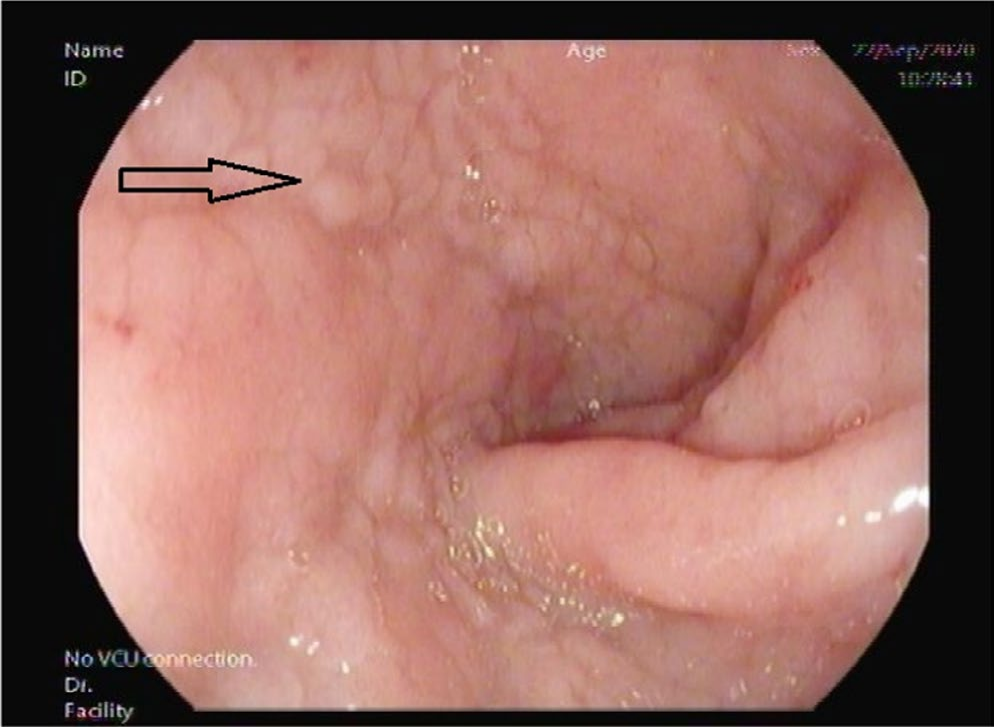
Duodenal bulb showing micronodular appearance (arrow)

**FIGURE 2:**
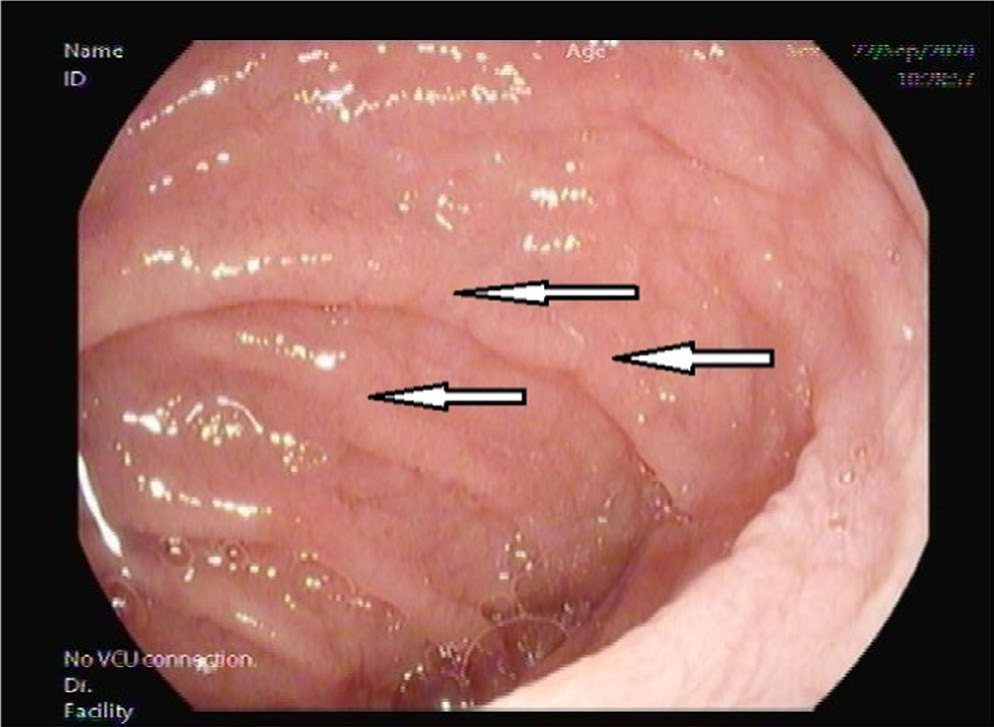
Second part of the duodenum endoscopic appearance shows a mosaic‐like pattern and scalloping of the mucosal folds (white arrows)

She had no complaints apart from occasional mild upper abdominal discomfort, and celiac disease screening revealed anti‐tissue transglutaminases IgA +50.00 U/ml anti‐tissue transglutaminases IgG 5.80 U/ml, anti‐endomysial IgA+, with IgA 3.41 gm/L(0.7–4 gm/L). She was not following any specific diet. Our institute performs combined Anti TTG IgA/igG routinely. An Esophagogastroscopy showed mosaic pattern with fold scalloping in the duodenal bulb and the second part of the duodenum (D1, D2) features consistent with celiac disease (Figures 1, 2).^10^, ^11^ Biopsies taken from D1 and D2 as per celiac protocol confirmed celiac disease, showing blunted, and flattened villi, increased intraepithelial lymphocytes, increased lamina propria plasmolymphocytic infiltrate. No granulomas, no Giardia, or malignant lesions were identified. After diagnosis, the patient was educated regarding the gluten‐free diet and scheduled for regular follow‐up.

## 3 DISCUSSION

Reactive thrombocytosis can be observed in various inflammatory conditions, with blood loss, or hyposplenism because platelets function as acute‐phase reactants.^12^ Work‐up usually starts with excluding secondary causes such as anemia and checking ferritin levels, which need to be corrected first. The degree of platelet count elevation is not related and cannot predict the underlying cause.^13^ Our patient's anemia was corrected with improvement in hemoglobin level and ferritin level but still patient's thrombocytosis did not improve. She had no features of infection or systemic illness, and the peripheral smear did not show features of hyposplenism, and work‐up for the secondary causes was performed. Next, MPN was to be excluded, and genetic tests were done (*JAK*‐2, *CALR*, *MPL*, and *BCR ABL* negative). She had a bone marrow examination which did not meet WHO criteria for other MPNs but had increased megakaryopoiesis. She was initially diagnosed with a triple‐negative disease based on bone marrow results. She had nonspecific upper abdominal symptoms for which celiac screen was performed and the result was positive. Adults are typically diagnosed with celiac disease with villous changes on duodenal mucosal biopsy in combination with positive serologic tests (anti‐tissue transglutaminase antibodies (tTG), anti‐endomysial antibodies (EmA), and deamidated gliadin peptide (DGP) antibodies). Pediatric patients can be diagnosed solely based on positive serologic test with high titers of antibodies with the requirement of mucosal biopsies.^11^

In celiac disease, platelet counts are usually elevated (rare to have thrombocytopenia), and it might be the only manifestation of celiac disease.^14^ The mechanism of thrombocytosis in celiac disease can be secondary to iron deficiency anemia due to malabsorption, leading to marked thrombocytosis,^15^ lymphoma, hyposplenism, or in other patients, no apparent explanation is found.^7^ This celiac disease related thrombocytosis might be the underlying cause of triple‐negative ET as some patients with celiac disease have varying clinical forms ranging from subclinical to refractory celiac disease. Using serological tests combined with duodenal biopsies confirmed that celiac disease's actual prevalence is much higher than expected.^16^

Compared with other mutations, triple‐negative disease patients are younger, have lower hemoglobin levels, lower leukocyte count, and lower incidence of thrombosis, but an elevated risk of blast transformation.^17^ With whole‐exome sequencing, only around 10.1% of patients with triple‐negative disease had identified somatic or germline mutations.^18^ This triple‐negative disease represents a heterogeneous group of disorders with natural history, management, and response to therapy unclear. The ambiguity of triple‐negative ET might mean that the underlying causes can be unidentified mutated genes (which may require whole genome sequencing) or other mimickers with close characteristics. Moreover, celiac disease patients have an increased risk of complications such as osteoporosis, malignancies including small bowel adenocarcinoma, Enteropathy associated T cell lymphoma, non‐Hodgkin's lymphoma, and mortality.^19^, ^20^ Interestingly, this patient had two failed pregnancies, which might be related to celiac disease complications affecting fertility. She was not on a gluten‐free diet before the diagnosis.^21^

Our group is studying the unmet needs and unanswered questions in Philadelphia negative MPNs such as priapism^22^ as well as unmet needs in CML such as the effects of intermittent fasting^23^ and obesity^24^in response to TKIs as well as priapism.^25^

In conclusion, patients with suspected essential thrombocythemia (ET) who are triple‐negative should be screened for celiac disease before doing more sophisticated studies such as gene sequencing or more invasive tests such as bone biopsy. The celiac screening will reveal underdiagnosed celiac patients, prevent celiac‐related complications, avoid expensive investigations, and avoid starting the patients on cytotoxic medication.

## CONFLICT OF INTEREST

All authors have no conflict of interest.

## AUTHOR CONTRIBUTIONS

Elrazi A. Ali, Kamran Mushtaq, Elabbass Abdulmahmuod, and Mohamed Yassin involved in writing editing and final approval.

## ETHICAL APPROVAL

This case report was approved by Hamad medical corporation research center and consent was taken from the patient.

## CONSENT

Written informed consent was obtained from the patient for publication of this case report.

## Data Availability

Data available on reasonable request.
